# Tides regulate the flow and density of Antarctic Bottom Water from the western Ross Sea

**DOI:** 10.1038/s41598-023-31008-w

**Published:** 2023-03-08

**Authors:** Melissa M. Bowen, Denise Fernandez, Arnold L. Gordon, Bruce Huber, Pasquale Castagno, Pierpaolo Falco, Giorgio Budillon, Kathryn L. Gunn, Aitana Forcen-Vazquez

**Affiliations:** 1 School of Environment, University of Auckland, Auckland, New Zealand; 2 NIWA, Wellington, New Zealand; 3 Lamont-Doherty Earth Observatory, Columbia University, New York, USA; 4 University of Messina, Messina, Italy; 5 Università Politecnica Delle Marche, Ancona, Italy; 6 University Parthenope, Naples, Italy; 7 CSIRO Oceans and Atmosphere, Hobart, TAS Australia; 8 LifeWatchERIC, Seville, Spain

**Keywords:** Physical oceanography, Projection and prediction

## Abstract

Antarctic Bottom Water (AABW) stores heat and gases over decades to centuries after contact with the atmosphere during formation on the Antarctic shelf and subsequent flow into the global deep ocean. Dense water from the western Ross Sea, a primary source of AABW, shows changes in water properties and volume over the last few decades. Here we show, using multiple years of moored observations, that the density and speed of the outflow are consistent with a release from the Drygalski Trough controlled by the density in Terra Nova Bay (the “accelerator”) and the tidal mixing (the “brake”). We suggest tides create two peaks in density and flow each year at the equinoxes and could cause changes of ~ 30% in the flow and density over the 18.6-year lunar nodal tide. Based on our dynamic model, we find tides can explain much of the decadal variability in the outflow with longer-term changes likely driven by the density in Terra Nova Bay.

## Introduction

**Figure 1:**
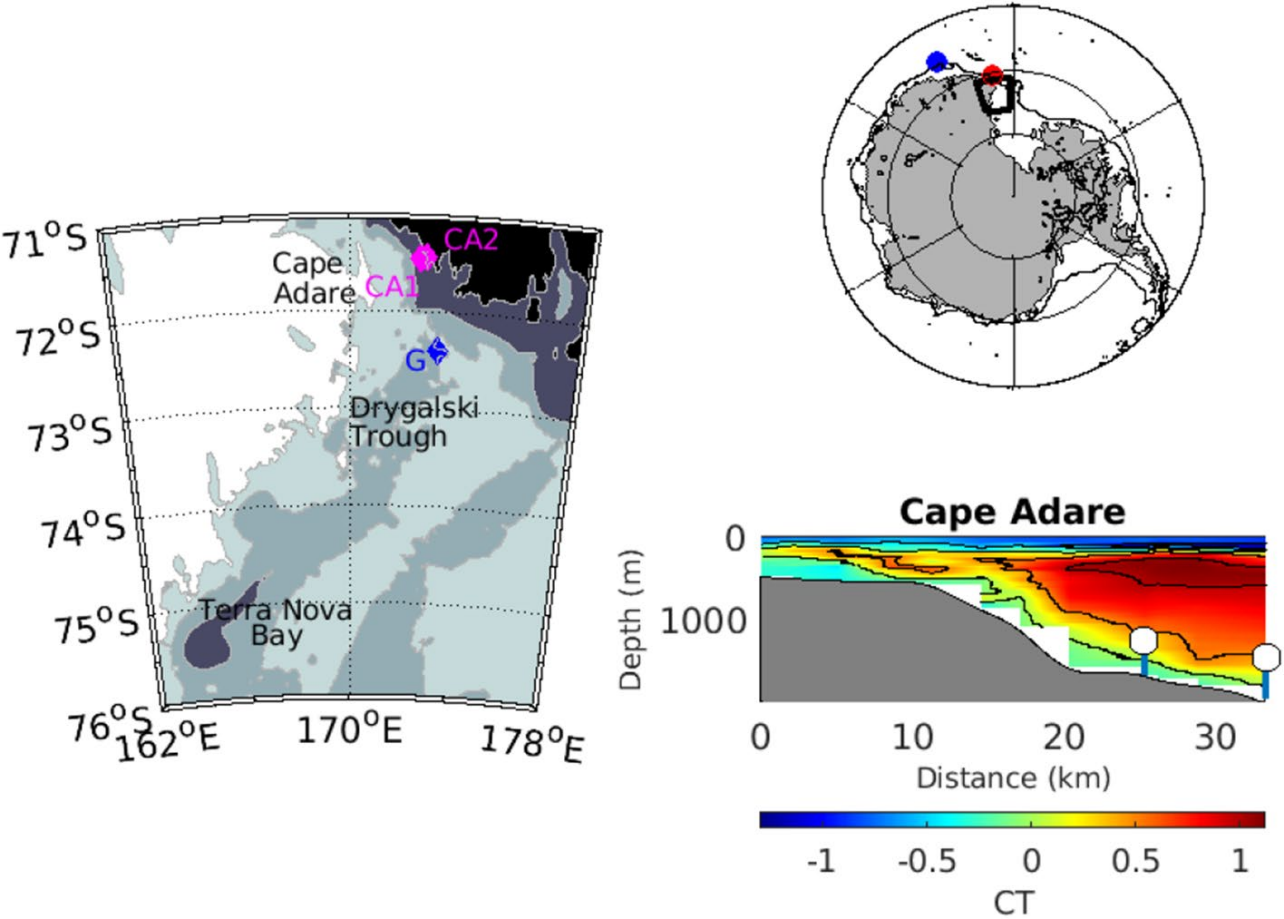
Observations of the dense outflow from the western Ross Sea (Left) Map of the western Ross Sea with the two mooring locations at Cape Adare (CA1/P2 and CA2) indicated by magenta diamonds and Mooring G in the Drygalski Trough indicated by a blue diamond. Depth contours are at 500, 1000 and 2000 m. (Top right) Outline of the western Ross Sea map inset is shown in black with the locations of the hydrographic observations at 170°E indicated by the red circle and 150°E by the blue circle. (Bottom right) The positions of the two moorings at Cape Adare (CA1 and P2 at the shallower depth; CA2 at the deeper depth) measuring benthic flow on the slope are shown with conservative temperature from the 2018 hydrographic section.

The properties of AABW set the temperature and salinity of approximately 40% of the global ocean^1^, determining much of the abyssal stratification^2^, rate of oxygen supply^3^ and CO_2_ uptake in the deep global ocean^4^. Dense water from the western Ross Sea, which exits the region at Cape Adare^5^ (Fig. 1), is the source of about a quarter of the global volume of AABW^6^. Hydrographic observations show AABW has freshened over the past few decades until 2014, with the greatest freshening near the Antarctic continent^7^ and slope^8^. Since 2014, salinity in the Ross Sea has increased^9^, as has salinity in the dense outflow at Cape Adare^10^, suggesting this component of the AABW may reduce or even reverse freshening trends in the deep ocean. Recent salinity increases in the Ross Sea, however, may be a shorter-term variation within the longer-term freshening trend observed from the 1950s^11^.

Winds are thought to cause variability in the production and release of dense water from the western Ross Sea. Increases in density in the Terra Nova Bay polynya are correlated with sea ice production and linked to lower import of sea ice from the east, suggesting a large-scale link between wind anomalies, dense water production, and AABW export^12^. Winds have also been proposed as the mechanism allowing release of dense water from the Drygalski Trough by moving the density fronts at the mouth^5^. In the Weddell Sea, dense water export is also correlated with changes in the winds and wind stress curl over the Weddell Sea Gyre^13^.

The northwestern Ross Sea, where the Drygalski Trough is located, also has exceptionally strong tides^14^ that likely influence the flow of dense water off the shelf. Observations document advection of dense water with the tides near bottom in the Drygalski Trough^15^ and mixing of modified Circumpolar Deep Water (mCDW) to near bottom during solstices^16^. Simulations also suggest tides control benthic layer properties^17,18^ and exchange through the trough^19^. Recent observations at Cape Adare show dense water pulses appear around equinox every year, consistent with weaker tides reducing bottom stress and allowing the release of dense water from the trough^10^.

Here we find the tides, together with the density of water in Terra Nova Bay, can explain much of the variability in the flow and density of dense water from the western Ross Sea. First, we show that flow and density from moored near-bottom measurements near Cape Adare are highly correlated with measurements of near-bottom flow in the Drygalski Trough. We then show that much of the variability in the flow in the trough is consistent with a balance between the bottom stress due to the tides and the pressure gradient along the trough created by the dense water in Terra Nova Bay. We find that large variations in the outflow twice a year may be caused by the tides varying with the declination of the Sun, and we suggest longer variations due to the 18.6-year lunar nodal cycle may also be present. We show that the modulation of the dense water flow and density is consistent with observed changes in salinity and thickness of the AABW layer downstream. Thus, we propose that tides are regulating the amount and density of AABW leaving the western Ross Sea and entering the global abyssal ocean.

## Results

### Dense water properties at Cape Adare

**Figure 2:**
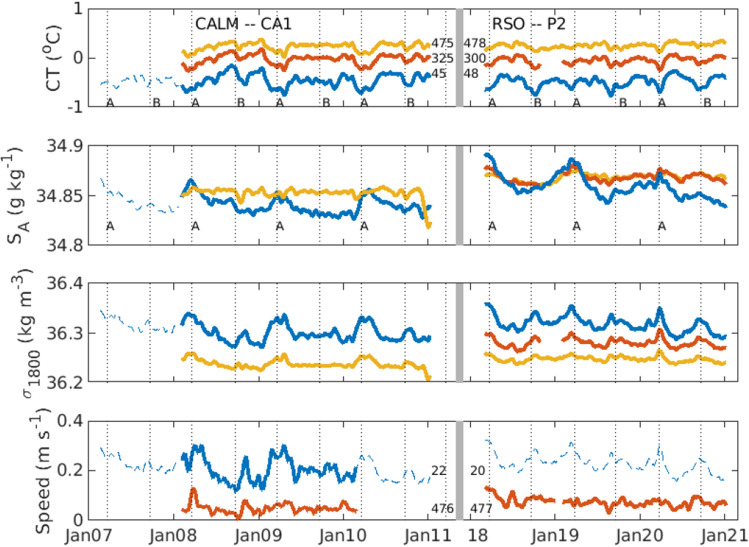
Observations of the dense water outflow at Cape Adare. Conservative temperature, absolute salinity, density referenced to 1800 db, and the flow speed on the slope at 1740 m depth near Cape Adare from sensors on the CA1 and P2 moorings. The height of the sensors above bottom is given by the black numbers in the panels. Dashed lines show a reconstruction of water properties and flow at the lowest sensor from the measurements at the nearby CA2 mooring in 2007 and a reconstruction of the flow from the density at the lowest sensor during the RSO deployments. Tick marks show January of every year with the grey bar indicating a discontinuity in time between the two deployments. Vertical dotted lines indicate the two equinoxes each year.

We first examine multiple years of moored observations at Cape Adare: these measurements show flow speed, temperatures, and salinities vary in a regular manner every year (Fig. 2). Temperatures have two colder periods each year (marked by ‘A’ and ‘B’) which are particularly evident between 2018 and 2021. The temperature minima correspond with equinoxes (vertical dotted lines) when the solar diurnal tides disappear, suggesting tides control the release of dense water from the Drygalski Trough. Salinity has one annual maximum in March (marked by ‘A’) coincident with colder temperatures. The annual maximum in salinity is consistent with the approximately 8-month time for dense water from Terra Nova Bay to advect to the mouth of the trough^10,20^ for release when tides are weak. Density has two maxima a year near equinoxes with higher density at March equinoxes when the water is saltier (see also Figure S1). Near-bottom density, measured at the sensor 45 m above bottom, is highly correlated with near-bottom flow, measured at the sensor 22 m above bottom (r = 0.87, *p* < 0.001, N = 21), as expected in a buoyancy-driven, geostrophic current.

Salinity and density of the bottom water exiting at Cape Adare also vary over the 15 years spanned by the observations (Fig. 2). Salinity decreased slightly between 2007 and 2011 during the CALM mooring deployments (a trend of − 0.003 yr^−1^), increased markedly between 2011 and 2018 when the mooring site was unoccupied (an increase of 0.027 between the last full year of the CALM experiment and the first full year of the RSO experiment), and decreased from 2018 to 2021 during the later mooring deployments (a trend of − 0.009 yr^−1^). These changes at Cape Adare are consistent with the salinity changes observed in the Ross Sea where a slow decrease in salinity was observed from 2007 until 2014 after which the salinity rebounded^9^.

### Dense water in the Drygalski Trough

**Figure 3:**
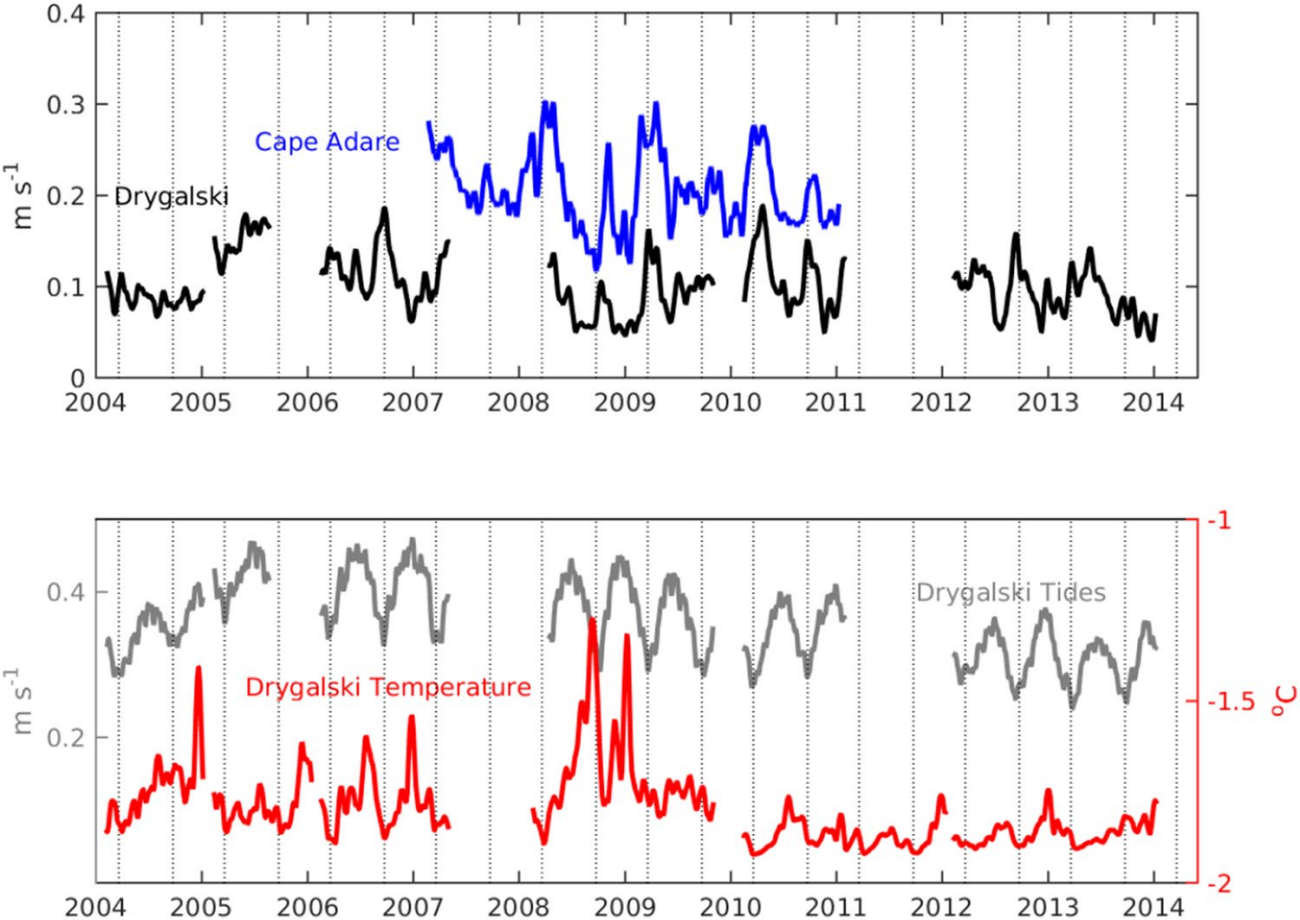
Water properties at Cape Adare and the Drygalski Trough (Top) Monthly-averaged, near-bottom flow at Cape Adare (blue) and in the Drygalski Trough (black) often increase at equinoxes (vertical dotted lines). (Bottom) The tidal flow in the Drygalski Trough, derived by subtracting the subtidal velocities from the full velocities, has a marked variation with lowest tidal flows during equinoxes (vertical lines) and highest tidal flows during solstices. Conservative temperature near bottom in the Drygalski Trough (red) increases during solstice when tidal velocities are highest.

The likely origin of the dense water at Cape Adare is the nearby Drygalski Trough. Water would reach the mooring location in less than a week after exiting the trough at the flow speed measured at Cape Adare. We find the monthly-averaged, near-bottom flow at Cape Adare is highly correlated with near-bottom flow measured at the mooring in the Drygalski Trough (r = 0.71 at zero lag, *p* = 0.0014, N = 32; Fig. 3), suggesting advection of water from the Drygalski Trough is the main source of the dense water observed at Cape Adare. The monthly-averaged flow in the Drygalski Trough can also explain the monthly-averaged fluxes of temperature and salinity at Cape Adare (Figure S2), and fluxes due to correlations of flow and water properties are negligible. Understanding what controls the flow in the Drygalski Trough is therefore necessary to understand the flow and density of bottom water at Cape Adare.

The observations in the Drygalski Trough show flows and temperatures vary markedly with the tides (Fig. 3). The monthly-averaged flow increases when the tidal flow decreases, particularly during equinoxes when solar diurnal tides disappear entirely. During solstices, tidal flows are maximum in the Drygalski Trough and warmer temperatures show mCDW is mixed down to near bottom (Fig. 3, lower panel, red line; see also ref^16^). Variations of the tidal flow in the Drygalski Trough change the monthly-averaged flow and temperature of the dense water near the bottom in the trough. These variations of flow and density create similar changes in the flow and density at Cape Adare.

**Figure 4:**
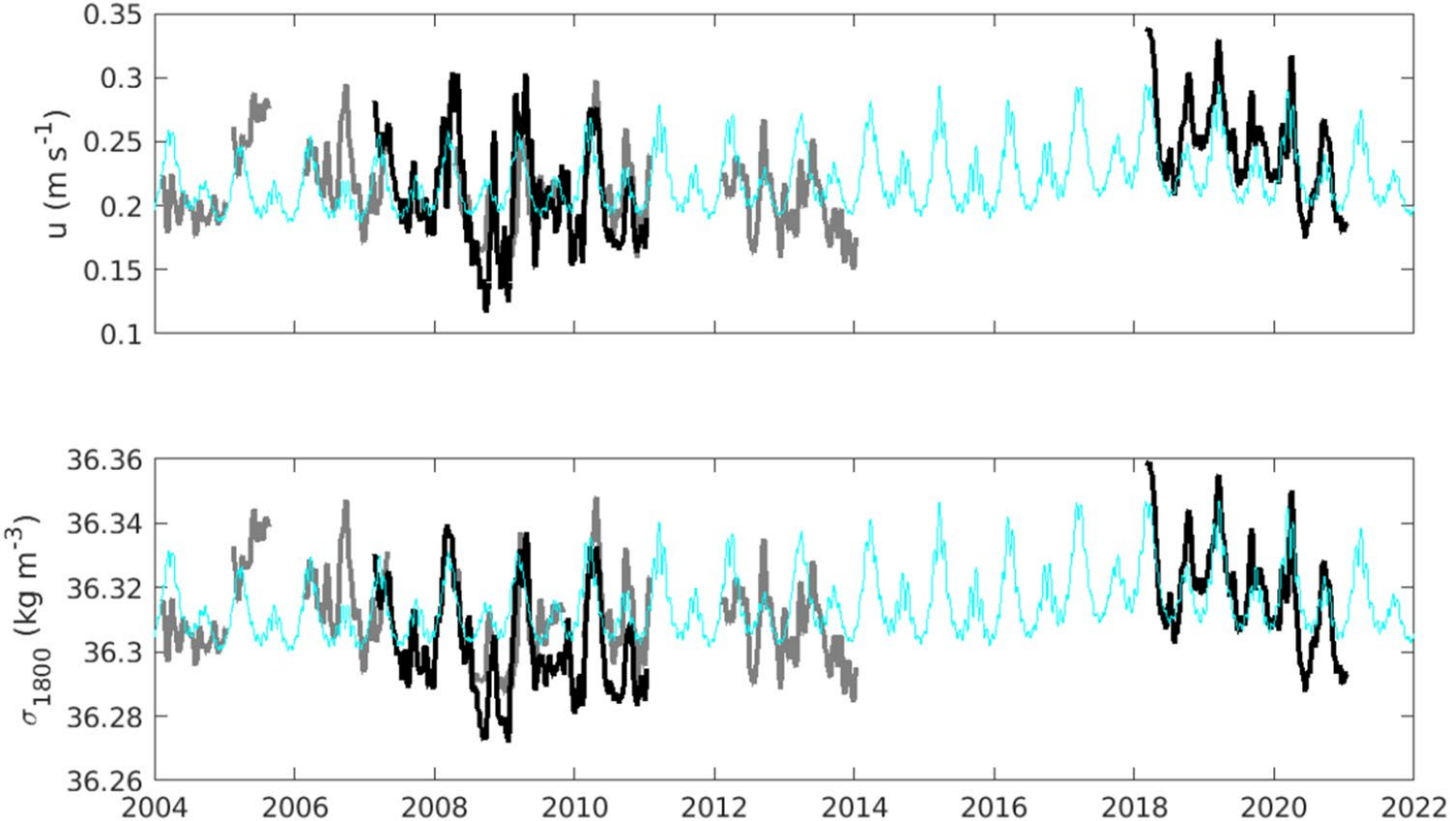
Observed and simulated flow and density at Cape Adare. Flow at the bottom sensor at Cape Adare (top) from measurements at Cape Adare (black lines), inferred values at Cape Adare using observations in the Drygalski Trough (dark gray lines) and simulated velocities at Cape Adare based on tides in the Drygalski Trough and density in Terra Nova Bay (blue line). (Bottom) Density at the Cape Adare mooring from the same set of observations and simulation.

We relate the monthly-averaged flow in the Drygalski Trough to the tides and dense water production in Terra Nova Bay using a momentum balance in the trough. We assume the flow at monthly time scales is due to a balance between bottom stress and pressure gradients along the trough (see methods for details). The pressure gradients are due to the sea surface slope and the density gradient along the bottom of the trough. We estimate the density gradient in the trough using a fixed density on the slope at the mouth of the trough and the time series of density in Terra Nova Bay^9^, varying it seasonally (Figure S3) consistent with observations in Terra Nova Bay at the depth of the trough^21^ and in the Drygalski Trough^16^. Using the linear relationships between the flow in the Drygalski Trough, the flow at Cape Adare and the density at Cape Adare (Figure S4) we reconstruct the monthly-averaged flow and density at Cape Adare (Fig. 4). The monthly-averaged velocity and density (Fig. 4, blue lines) contains much of the semi-annual, annual, and interannual variability observed (Fig. 4, dark grey lines; r = 0.72, *p* < 0.001, N = 32; Figure S5 compares the interannual variability) but does not capture some extreme values in the observed time series.

### Dense water export varies with tides in the Drygalski Trough and salinity in Terra Nova Bay

**Figure 5:**
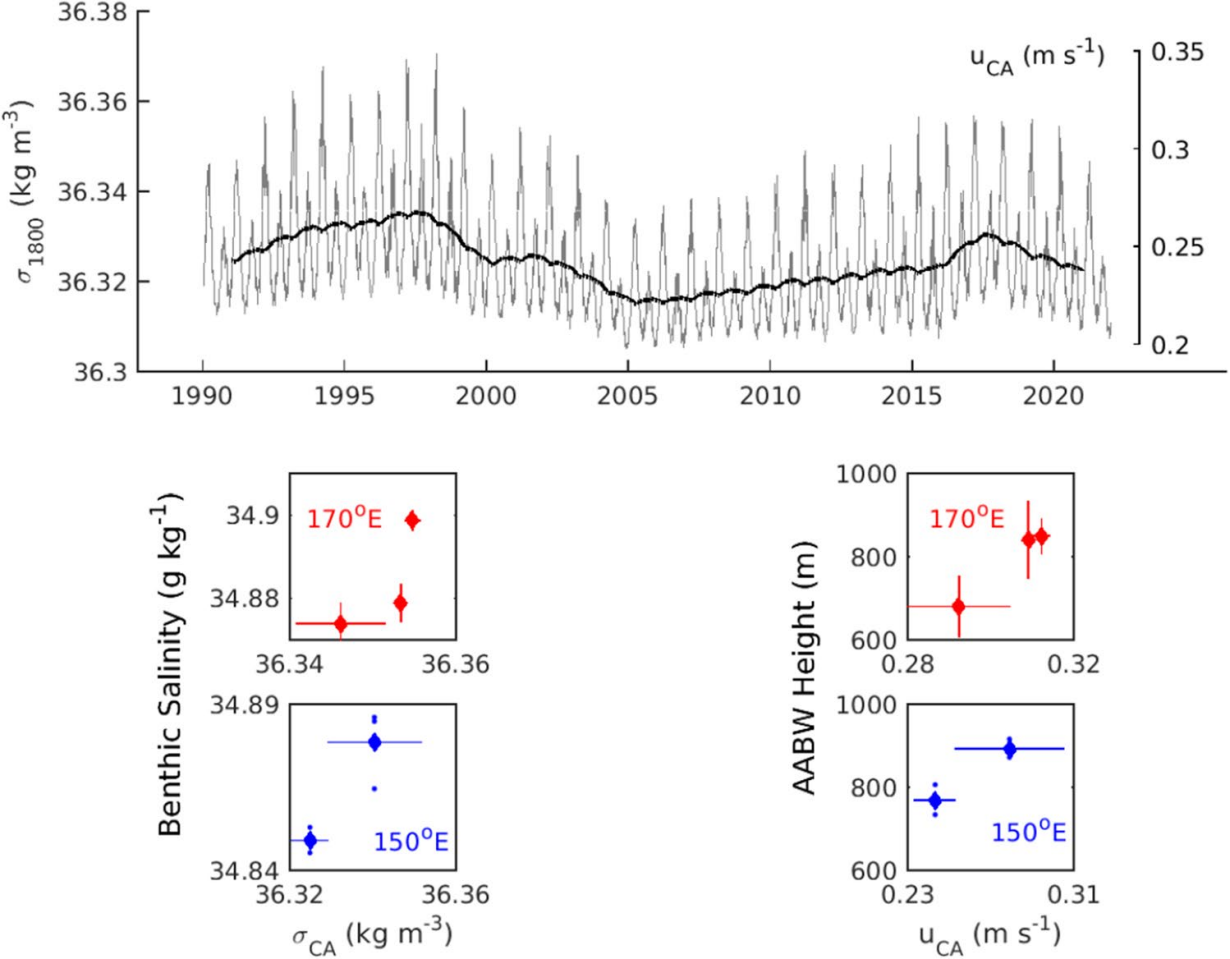
Simulated flow and density of the dense outflow at Cape Adare. (Top) Density and flow at Cape Adare estimated from the tides in the Drygalski Trough and density in Terra Nova Bay. The running average over two years is plotted in black to show the interannual variability. (Bottom left panels) Absolute salinity averaged over the bottom 300 m from hydrography at 170°E (red) and 150°E (blue) is plotted against the estimated outflow density. (Bottom right panels) The height of the 0 °C above bottom in the hydrographic sections at 170°E (red) and 150°E (blue) is plotted against the estimated outflow velocity. See methods for details of the averaging and uncertainty estimates.

Using the relationship between the outflow, the tides, and the density in Terra Nova Bay, we infer the flow and density of dense water at Cape Adare from 1990 to 2022 (Fig. 5). The interannual variation in the simulated flow and density are produced by a combination of the tides and the salinity changes in Terra Nova Bay. The strongest and most dense outflow occurs around 1997 when the salinity in Terra Nova Bay is high and the tides are weakest during a minor lunar standstill (Figure S3). The weakest, least dense outflow occurs near 2005 during a major lunar standstill when tides are strongest. Our model suggests most of the decrease in the outflow between 1997 and 2005 is due to the tides (Figure S3), as is most of the increase in the outflow from 2005 to 2015, with the change in density only contributing since 2015. In the simulated outflow, the tides have been responsible for much of the modulation of the velocity and density on decadal time scales. Between the two occupations of the mooring site at Cape Adare, the tides and density have contributed about equally to the increase.

We compare the prediction of dense water outflow from the western Ross Sea with hydrographic observations in the outflow further west on the slope at 170°E (120 km from Cape Adare), and at 150°E (1200 km from Cape Adare) (Fig. 1), where salinities have freshened since the early 1990s and become saltier in recent years^12,22^. Benthic salinities and the height of the AABW layer increase at both locations when the outflow density and flow increase (Fig. 5, bottom panels). The measurements of AABW downstream of Cape Adare are consistent with the variability in the estimated strength and density of the outflow from the western Ross Sea. We note, however, that the observations are limited in capturing decadal variability; these results are not conclusive but do show the dynamic model is plausible.

## Discussion

**Figure 6:**
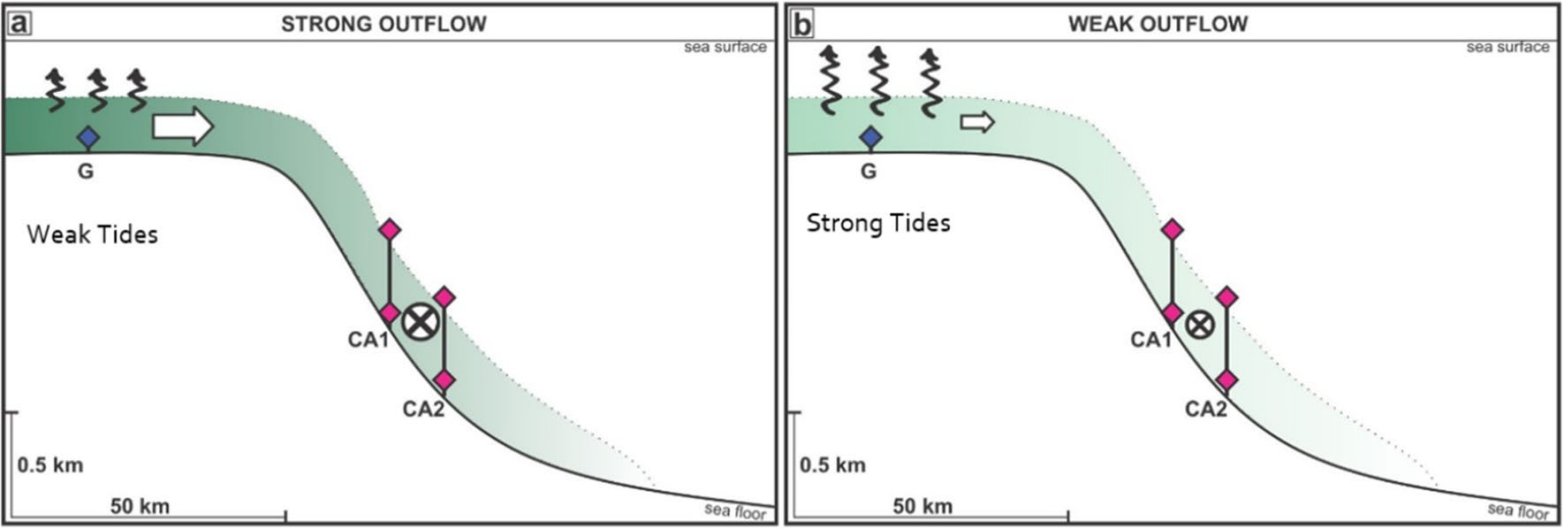
The link between the outflow, the tides and the density in Terra Nova Bay. The flow out of the trough (white arrows) turns to the left and flows along the slope past the moorings (flow is into the page in the diagram and indicated by the circle with a cross). The strength of the outflow depends on the tidal mixing in the trough (curly arrows) and the density of the bottom water from Terra Nova Bay (shown by the green shading where darker green indicates denser water). (**a**) A stronger outflow of more dense water results when tides are weaker and water in Terra Nova Bay has a higher density. (**b**) A weaker outflow of less dense water results when tides are stronger and water in Terra Nova Bay is less dense.

We explain the flow and density of bottom water exiting the western Ross Sea as a balance between the density in Terra Nova Bay at the depth of the trough driving the outflow and the bottom stress due to the tides in the trough slowing the flow down (Fig. 6). The tides in the Ross Sea change markedly because they are predominantly diurnal and vary with the declination of the Sun and Moon, disappearing when the Sun and Moon are at the Equator. The energy available for mixing due to shear production (proportional to the cube of the tidal velocity) in the Drygalski Trough reduces by 45% on average from solstice to equinox and by 40% from major to minor lunar standstill over the 18.6-year lunar nodal cycle. Sensitivity to these large changes in tidal energy in the Ross Sea should be considered in simulations of dense water formation and the outflow. More conclusive statements about the lunar nodal tide will require longer time series: locations with multi-decadal observations do show changes with the lunar nodal tide in ocean temperatures^23^, particularly in places where diurnal tides are strong, such as the California coast^24^ and at high latitudes^25^. Short observational records from around the Antarctic report tidal fluctuations in overflows from the Weddell Sea^26^ and in circulation within ice shelf cavities^27,28^: our study suggests the 18.6-year lunar tidal cycle may modulate horizontal flows that move heat across the shelf and under ice sheets.

The change of the outflow by the tides suggests the compensating inflow of water to the shelf is also modulated by the tides. An inflow of mCDW brings heat, salt, and nutrients into the Ross Sea and likely also varies markedly through the year and over the decades with the tides. We can estimate the contribution to the salt balance by estimating the dense outflow as 0.2 Sv on average over the year based on the previous estimate of the peak dense outflow of 0.4 Sv^5^. If this outflow is replaced by an inflow of 0.1 g kg^−1^ less salty water^16^, a variation of 30% over a decade (the magnitude of changes seen in the simulated flow in Fig. 5) is equivalent to a change of 20 km^3^ yr^−1^ of ice import. This change in salt is smaller than the 65–150 km^3^ yr^−1^ of ice export estimated to be caused by the winds^12^, suggesting the exchange flow in the trough is a smaller contribution to the salt balance than sea ice changes, but would still be a substantial factor in the Ross Sea salt balance.

Our study also shows that the vertical distribution of salinity in the Ross Sea is important for the outflow. The outflow in the model is driven by the salinity at 500 m depth in Terra Nova Bay, the depth of the bottom of the Drygalski Trough. The annual appearance of fresher water reduces this density every year^21^ and the salinity of the water flowing out of the trough^16^. Whether the Drygalski Trough continues to export dense water to the global ocean or whether it flips to an inflow of warmer water towards the Ross Ice Shelf depends critically on the salinity at 500 m depth in Terra Nova Bay.

## Methods

### Moored observations

Mooring observations were collected at two sites on the slope at Cape Adare spanning seven years in total, in the CALM experiment (2007–2011)^5^ and the Ross Sea Outflow Experiment (2018–2019)^10^ and the recent continuation in the New Zealand Antarctic Science Platform (2019–2021). The records were averaged over a month using a cosine filter of 29 days. The longest time series was collected at the shallower site, named CA1 and P2 depending on the experiment, located at 172.30°E, 71.46°S in 1740 m depth. All sensors were calibrated before and after the deployments and any differences applied linearly over the record.

Monthly-averaged densities at the near-bottom sensor at the shallower mooring site were reconstructed in 2007 (Fig. 2, dashed lines), when the deeper site (CA2; 172.39°E, 71.43°S, 1920 m) was the only mooring, using the relationship between the near bottom density at CA2 and CA1 during the overlapping years (r = 0.98, *p* < 0.001, N = 30; Figure S4). (Figures of these two mooring time series are presented in reference^5^.) We use the speed of the monthly-averaged velocities at the lower sensor because the direction is always northeastward with little variation (the mean direction is 57° north of east and 80% of the time the velocity is within 15° of the mean direction). The monthly averages of density and speed are also highly correlated at the near-bottom sensors (r = 0.87, *p* < 0.001, N = 21; Figure S4) at CA1, and we use this relationship to reconstruct the speed when the current meters failed.

Moored observations were collected in the Drygalski Trough between 2004 and 2014 at Mooring G near 72.4°S, 173°E in approximately 520 m water depth as part of the MORSea experiment^16^. Observations of temperature and velocity were used from near-bottom sensors. The velocities were first adjusted to 30 m above bottom using a log layer scaling to mitigate the effects of the sensor depth changes between deployments. The diurnal tides were removed from the velocities by averaging the components over three days with a cosine window. We use the magnitude of the velocity to infer the magnitude of the near-bottom flow in the trough. The tidal velocities, which contain the diurnal variations, were found by subtracting the three-day averages from the full velocity components.

### Drygalski Trough momentum balance

The monthly variation of flow in the trough is related to the tides and density in the trough using a momentum balance along the axis of the trough (the x-direction where the direction towards the ocean is positive; see schematic in Figure S6):

$$\frac{\partial u}{{\partial t}} + u\frac{\partial u}{{\partial x}} + v\frac{\partial u}{{\partial y}} + w\frac{\partial u}{{\partial z}} - fv = - \frac{1}{{\rho_{0} }}\frac{\partial p}{{\partial x}} + \frac{1}{{\rho_{0} }}\frac{{\partial \tau^{x} }}{\partial z}$$

where only the stress divergence in the vertical has been retained. Integrating the balance over a vertical distance (h) from the sea floor to the top of the dense water layer, where we assume stress is small, the total stress is primarily due to the bottom stress, which can be related to the velocity (u) with a drag coefficient (C_D_).

$$h\frac{\partial u}{{\partial t}} + N - f\mathop \smallint \limits_{0}^{h} v dz = - gh\frac{\partial \eta }{{\partial x}} - \frac{{gh^{2} }}{{2\rho_{0} }}\frac{\partial \rho }{{\partial x}} - C_{D }\, u \left| u \right|$$

The pressure gradient has been separated into a part due to the slope of the sea surface ($\eta$) and a part due to the along-trough density gradient ($\partial \rho /\partial x)$ which we assume is constant with depth in the dense water layer. The integrated non-linear terms are represented by N. We now average each term in time to find the monthly-averaged momentum balance using the subscript “s” to represent the monthly-averaged quantities.

$$h\frac{{\partial u_{S} }}{\partial t} + N_{S} = - gh\frac{{\partial \eta_{S} }}{\partial x} - \frac{{gh^{2} }}{{2\rho_{0} }} \frac{{\partial \rho_{S} }}{\partial x} - C_{D }\, u_{S} \left| u \right|$$

We have simplified the stress by noting that the tidal velocities are considerably larger than the monthly-averaged velocities (u >  > u_s_). Additionally, the Coriolis term has been ignored because geostrophic flows perpendicular to the sides of the trough should be small. The depth- and time-averaged non-linear terms (N_S_) are unknown. We would expect these terms to scale with the tidal velocities and some length scale (~ hu_tide_^2^/L); however, the observed monthly-averaged flow decreases with increasing tidal velocities (Fig. 3), thus we infer that the non-linear terms are not a dominant contribution to the monthly-averaged momentum balance. We also assume the time rate of change of the monthly velocities is small compared to the bottom stress. With these assumptions, the balance is between the pressure gradients (created by the slope of the sea surface, η_S_, and the gradient of the density, ρ_S_, along the trough) and the bottom stress which can be rearranged as an expression for the monthly-averaged velocity:

$$u_{S} = \frac{ - gh}{{C_{D} \left| {u_{tide} } \right|}} \left( {\frac{{\partial \eta_{S} }}{\partial x} + \frac{h}{{2\rho_{0} }}\frac{{\partial \rho_{S} }}{\partial x}} \right) \approx \frac{1}{{\left| {u_{tide} } \right|}}\left( {A + B{\Delta }\rho } \right)$$

The balance is simplified to two terms: the first is a constant, A, which represents a constant pressure gradient, and the second term, which contains a constant B multiplying the pressure gradient due to the density difference between Terra Nova Bay and the slope. We anticipate that much of the surface pressure gradient will be proportional to the density gradient to balance the net flow, as in an exchange flow^29^, but a residual pressure gradient is likely to be present to balance mass in the Ross Sea. We assume the stress is small at the top of the dense layer, but if the stress at the top of the layer is proportional to the bottom stress, the same relationship would hold. A more complex variation of stress through the water column, however, would not be captured well by this model.

We estimate the density gradient along the trough as the difference between a fixed density on the slope (chosen from hydrographic surveys to be S_A_ = 34.89 g kg^−1^ (Sp = 34.72) and θ = 0.5 °C) and density at the depth of the trough in Terra Nova Bay. The density in Terra Nova Bay is varied seasonally with an amplitude of 0.05 kg m^−3^ (Figure S3, center panel) consistent with observations both in the bay^24^ and in the Drygalski Trough^16^. The peak value of the salinity in the trough is in March and is set to the hydrographic measurements of the dense water in Terra Nova Bay the previous summer^9^, with additional hydrographic measurements from the World Ocean Database averaged in the same manner (as described below).

The coefficients, [A, B] = [0.02, 0.1], were determined by the best fit to the observations of monthly-averaged, near-bottom velocity in the Drygalski Trough and velocity in the trough inferred from the observations at Cape Adare (scaled by the linear relationship between the two locations). We hold the density on the slope fixed because we lack information about how it varies: however, we found adding terms proportional to the along-slope wind stress and Ross Gyre wind stress curl (to account for the movement of the density front at the mouth of the trough by local and remote winds) adds little additional skill to the fit.

### Tidal analysis

We estimate the tidal velocities in the Drygalski Trough over all time by fitting the diurnal (${\zeta }_{1}$) and low frequency (${\zeta }_{0}$) tidal constituents to the tidal velocities at Mooring G. We ignore the semidiurnal tide, which is small at high latitudes.

$$u_{tide} = a_{0} \frac{{\zeta_{0} }}{{10^{9} }} + a_{1} \frac{{\zeta_{1} }}{{10^{9} }} + a_{2}$$

The constituents at a latitude ($\theta$) on Earth have contributions from both the Sun and the Moon that change with the distance (r) between the celestial body and Earth and with the declination ($\delta )$ of the body from the Equator:

$$\begin{aligned} & \zeta_{0} = \frac{{mR^{4} }}{{4Mr^{3} }} (3\sin^{2} \theta - 1) (3\,\sin^{2} \delta - 1) \\ & \zeta_{1} = \frac{{mR^{4} }}{{4Mr^{3} }}\,\left( {3\,sin2\theta\,sin2\delta } \right) \\ \end{aligned}$$

where the mass and radius of the Earth are M and R and the mass of the celestial body is m.

We use the ephemerides from Jet Propulsion Laboratory Horizons Web-Interface^30^ to find the distances and declinations of the Sun and the Moon. We find coefficients when ${u}_{tide}=0$, which simplifies the fit and ensures weaker tides are captured when the advection of dense water is largest. We use the times when the weekly average of the velocity magnitude is less than 0.2 m s^−1^ as approximate times of no flow and find [a_0_, a_1_, a_2_] = [0.87, − 1, 0.95]. The comparison between the tidal velocities from the mooring and the fit is shown in Figure S3.

### Hydrography

Salinities of the dense water in Terra Nova Bay used to estimate the seasonal density creating the pressure gradient at the bottom of the trough (Figure S3) are from previous measurements (9). Three additional estimates were made for 1984, 1997, and 2007 from hydrography in the World Ocean Database and averaged in the same manner: salinities were averaged over 870 to 900 dbars from casts obtained 74.75°S–75.50°S and 163.00°E–166.00°E at stations with depths greater than 800 m.

Shipboard hydrography at 150°E and 170°E were obtained from the World Ocean Database collected in April 1993 and February 2018 at 150°E and March 1992, March 2011 and April 2018 at 170°E. Salinity over the bottom 300 m and the height of the AABW layer above bottom (from the height of 0 °C temperature) in each cast were calculated for comparison to the outflow density and velocity from the western Ross Sea (Fig. 5).

The surveys at 170°E are approximately 120 km from Cape Adare and, at the average flow speed observed of 0.2 m s^−1^, the water would take one week to travel between the two locations. We calculated the standard deviation of flow and density estimates at Cape Adare from all values between five and fifteen days before the date of each hydrographic survey. To find uncertainty of the salinity and height of AABW in the three surveys at 170°E, the standard error was computed by finding the standard deviation of the ten casts in each survey and dividing by the square root of ten.

The surveys at 150*°*E are approximately 1200 km from Cape Adare and, at 0.2 m s^−1^, the water takes about two months to travel between the two locations. We averaged the flow and density estimates at Cape Adare between 50 and 100 days before the date of the hydrographic sections and compute the standard deviation of the values within that time span to estimate uncertainty. The range of salinities and heights of AABW from the hydrography are shown by plotting the values from the three casts in each survey as blue dots.

### Statistical analysis

Correlation coefficients are reported with p-values based on N, the effective degrees of freedom, and account for the autocorrelation of each time series. N was determined by integrating the autocorrelation of each time series from the origin to the first zero crossing to estimate an integral time scale. The total time span of the data was divided by the integral time scale to give N. For each correlation, the smallest N of the three values (two from the autocorrelation of each time series and one assuming each month is independent) was used when determining the p-value.

## Supplementary Information

Supplementary Information.

## Data Availability

Hydrographic data from the sections east of the Ross Sea and Terra Nova Bay are available from the World Ocean Database. Additional hydrographic data from Terra Nova Bay are available from the authors. Moored data from the Drygalski Trough are available from the MorSea website and on request from the authors.
