# Author Correction: Tides regulate the flow and density of Antarctic Bottom Water from the western Ross Sea

**DOI:** 10.1038/s41598-023-32101-w

**Published:** 2023-03-27

**Authors:** Melissa M. Bowen, Denise Fernandez, Arnold L. Gordon, Bruce Huber, Pasquale Castagno, Pierpaolo Falco, Giorgio Budillon, Kathryn L. Gunn, Aitana Forcen-Vazquez

**Affiliations:** 1 School of Environment, University of Auckland, Auckland, New Zealand; 2 NIWA, Wellington, New Zealand; 3 Lamont-Doherty Earth Observatory, Columbia University, New York, USA; 4 University of Messina, Messina, Italy; 5 Università Politecnica Delle Marche, Ancona, Italy; 6 University Parthenope, Naples, Italy; 7 CSIRO Oceans and Atmosphere, Hobart, TAS Australia; 8 LifeWatchERIC, Seville, Spain

Correction to: *Scientific Reports* https://doi.org/10.1038/s41598-023-31008-w, published online 08 March 2023

The Funding section in the original version of this Article was omitted. The Funding section now reads:

“This study and support for M.B. and D.F. were funded by the New Zealand Strategic Science Investment Fund: Antarctic Science Platform Contract ANTA1801. A.F.-V. was supported by the Deep South National Science Challenge. The mooring G data were collected in the framework of MORSea projects supported by the Italian National Program for Antarctic Research‐PNRA, which provided financial and logistic support. P.C. was funded by the PNRA, Grant PNRA18_00256. The PNRA is funded by the Italian Minister of the University”.

The original Article has been corrected.

