# Supplementary material for: Tides regulate the flow and density of Antarctic Bottom Water from the western Ross Sea
Source: Sci Rep. 2023 Mar 8;13:3873. doi: 10.1038/s41598-023-31008-w (PMC9995308; doi:10.1038/s41598-023-31008-w)
Supplement: Supplementary file 1 — Supplementary Information. [file 41598_2023_31008_MOESM1_ESM.docx]

Supplementary Material

Tides regulate the flow and density of Antarctic Bottom Water from the western Ross Sea

**Authors**

Melissa M. Bowen, Denise Fernandez, Arnold L. Gordon, Bruce Huber, Pasquale Castagno, Pierpaolo Falco, Giorgio Budillon, Kathryn L. Gunn, Aitana Forcen-Vazquez


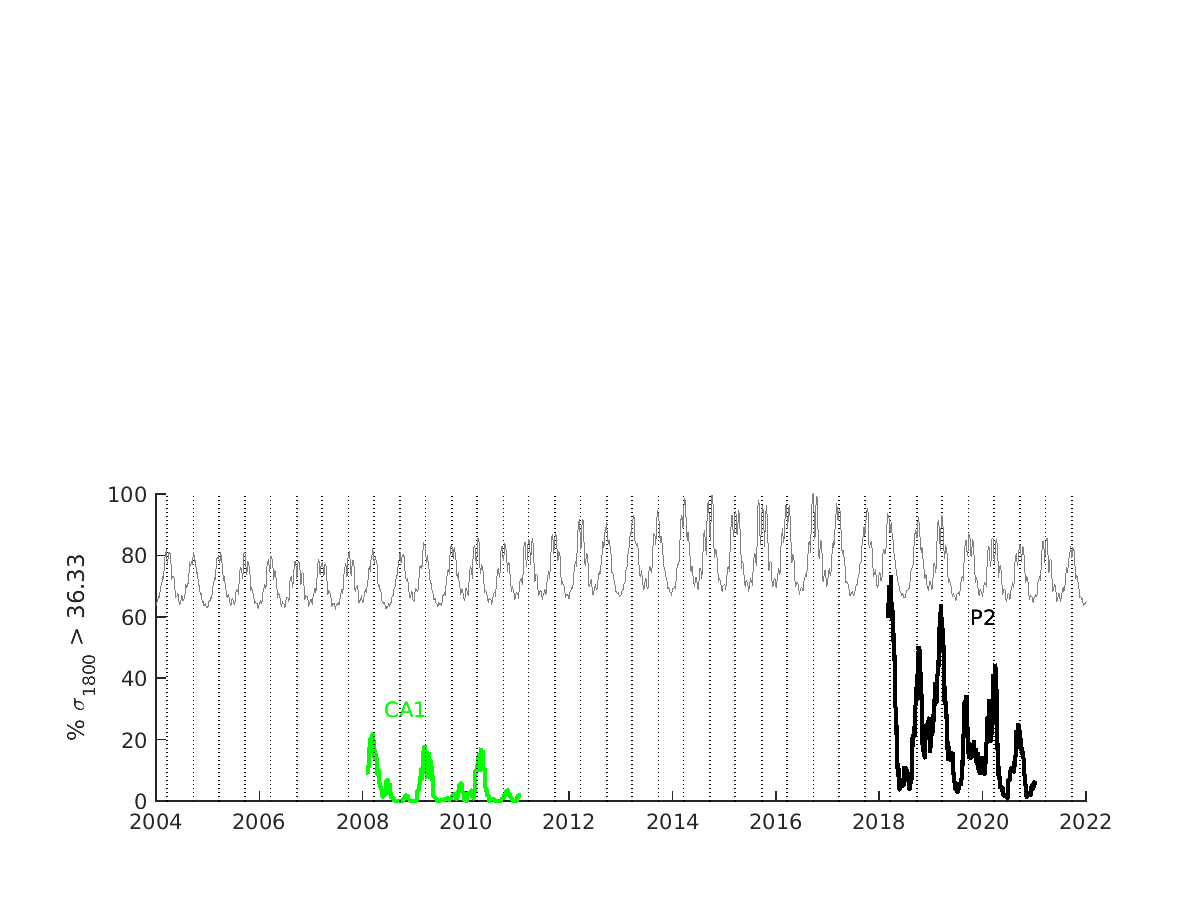


Fig. S1. The presence of dense water at the Cape Adare is shown by the percentage of time each week the density at the lowest sensor of the CA1 or P2 mooring is above 1036.33 kg/m^-3^. The gray line shows the inverse of the tidal velocity magnitude in the Drygalski Trough (from the tidal fit described in the methods) plotted on an arbitrary scale. The vertical dotted lines mark the equinoxes.


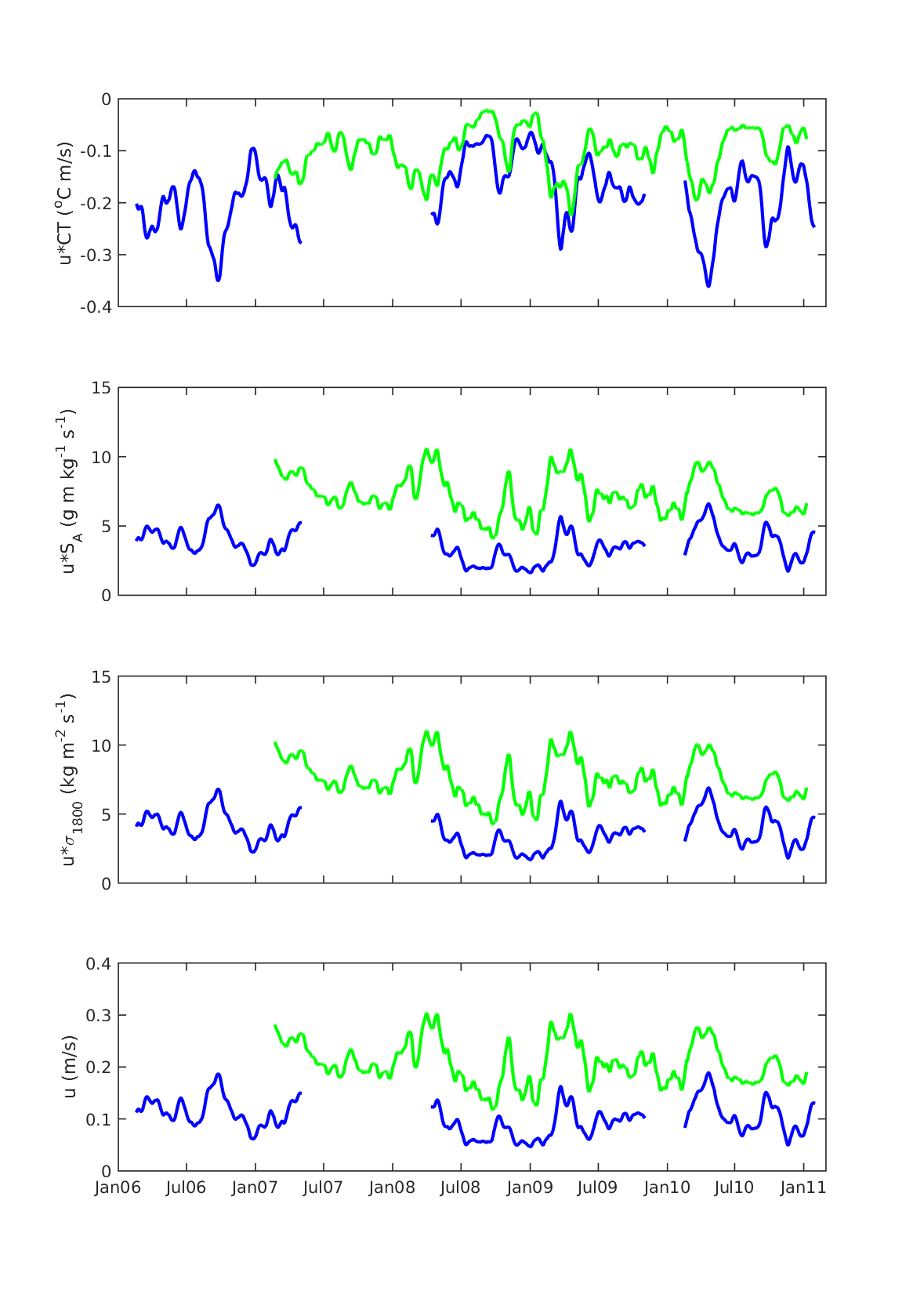
Fig. S2. Fluxes of conservative temperature, absolute salinity, and density at Mooring G (blue) and Cape Adare (green) from monthly-averaged quantities. The lower panel shows the monthly-averaged flow at the two moorings.


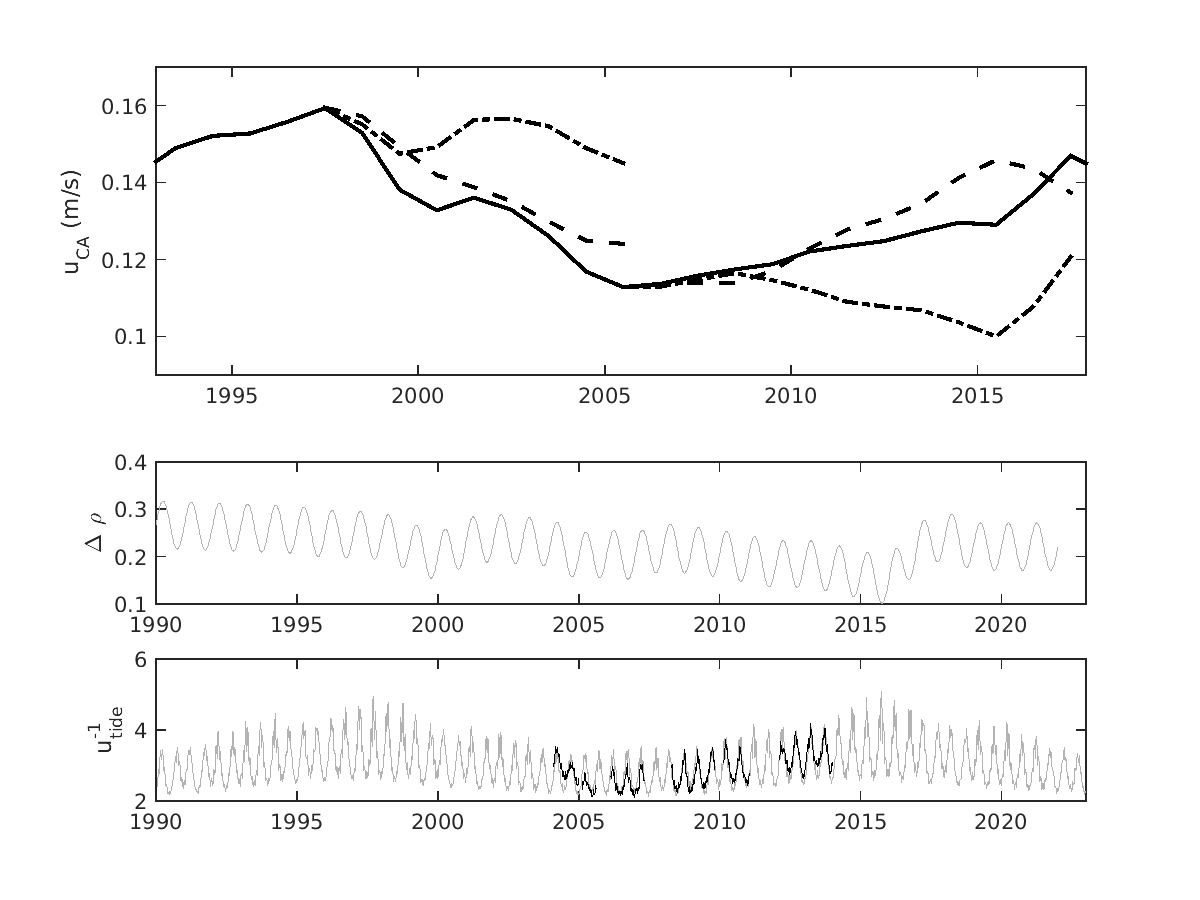


Fig. S3. The upper panel shows annually-averaged velocity at Cape Adare (black line) from the relationship based on the density difference between Terra Nova Bay and the slope (central panel) and the inverse of the tidal velocities in the Drygalski Trough (lower panel). The dashed lines show the simulated flow starting at two different times. In both cases the density gradient was held fixed and only the tides changed. The dashed dotted lines start at the same times and show the simulated flow with the tides held fixed and the density gradient changed with time. The tidal flow from the moored observations in the Drygalski Trough is plotted in black in the lower panel.


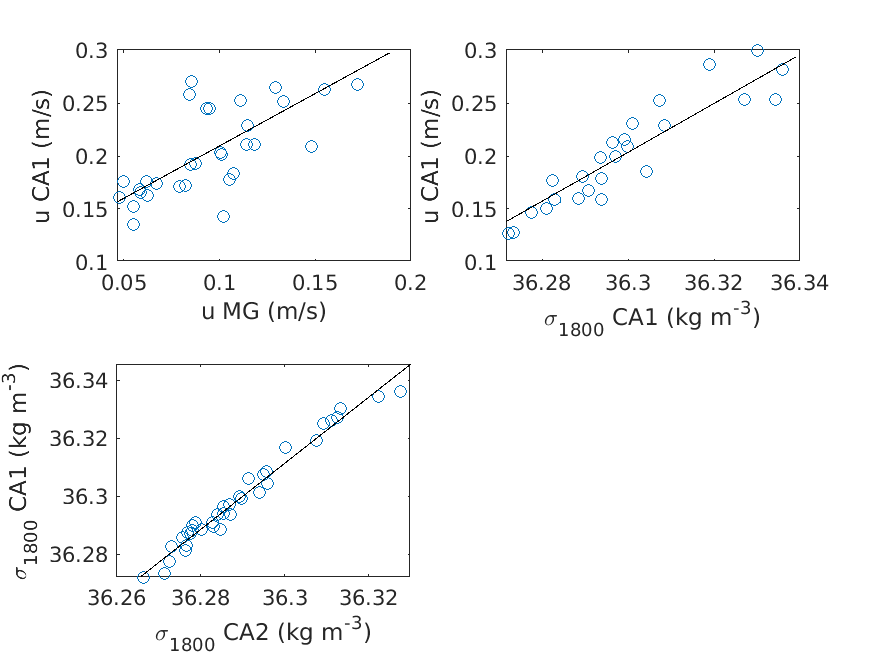


**Fig. S4.** The relationships between near-bottom, monthly-averaged velocities and densities at the moorings are shown in regression relationships: (top left) velocity in the Drygalski Trough at Mooring G (x-axis) and velocity at Cape Adare at CA1 (y-axis), (uCA1 = 0.99 uMG + 0.11; r^2^ = 0.50); (top right) density at Cape Adare at the CA1 (x-axis) and velocity at Cape Adare (y-axis), (uCA1 = 2.31 σCA1 – 83.48; r^2^ = 0.76); (bottom left) density at the C2 mooring at Cape Adare (x-axis) and density at the C1 mooring at Cape Adare (σCA1 = 1.14 σCA2 – 4.94; r^2^ = 0.96).


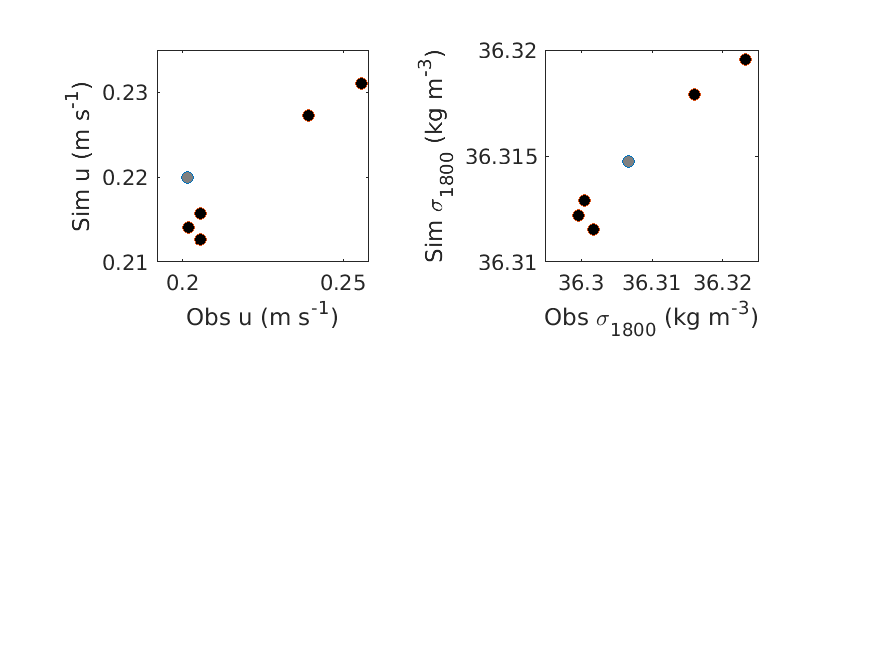


**Fig. S5.** Interannual variability in the simulated and observed velocity (left) and density (right) are compared using averages over two-year segments of data. The years with mooring data at Cape Adare are shown with black circles (three averages from the CALM experiment 2007-2009, 2008-2010, 2009-2011 and two averages from the RSO experiment 2018-2020, 2019-2021) and grey circles when the mooring in the Drygalski trough (2012-2014) was used to infer properties at Cape Adare.


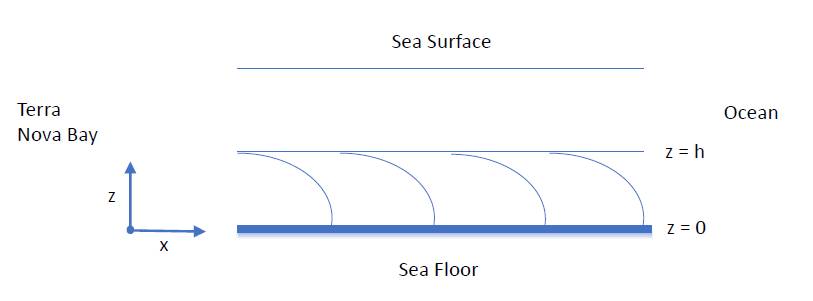


**Fig. S6.** Schematic of the momentum balance in the Drygalski Trough. The-x axis is oriented along the trough with the positive direction towards the ocean. The y-axis (not shown) is across the trough and positive into the page. The z-axis is upward with zero on the bed of the trough and the top of the dense water layer a distance h above the bed. The density increases towards Terra Nova Bay. The gradient in density is shown as isopycnals by the curved lines in the lower layer.
